# Endovascular resuscitation: an expert practice review

**DOI:** 10.1136/emermed-2025-215376

**Published:** 2026-04-30

**Authors:** Thomas David Adams, Jon Barratt, James E Manning, Paul Rees, Ben Singer, Ed Benjamin Graham Barnard

**Affiliations:** 1 Department of Research, Audit, Innovation and Development, East Anglian Air Ambulance, Norwich, Norfolk, UK; 2 Emergency and Urgent Care Research in Cambridge (EUReCa), PACE Section, University of Cambridge School of Clinical Medicine, Cambridge, England, UK; 3 Academic Department of Military Emergency Medicine, Royal Centre for Defence Medicine (Research and Clinical Innovation), Birmingham, Yorkshire, UK; 4 Yorkshire Air Ambulance, Elland, West Yorkshire, UK; 5 Emergency Medicine, The University of North Carolina at Chapel Hill School of Medicine, Chapel Hill, North Carolina, USA; 6 Shock Trauma Center, University of Maryland, Baltimore, Maryland, USA; 7 Barts Heart Centre, St Bartholomew’s Hospital, London, UK; 8 London’s Air Ambulance Charity, London, England, UK

**Keywords:** Out-of-Hospital Cardiac Arrest, pre-hospital care, critical care, Cardiopulmonary Resuscitation, resuscitation

## Abstract

Endovascular resuscitation (EVR) describes a collection of novel therapies that aim to bridge patients in shock states or cardiac arrest to definitive treatment, through a combination of haemorrhage control and mechanical circulatory support. The last half-decade has seen landmark trials report on several techniques and adoption is growing despite controversies around risk, efficacy and implementation. As leaders in resuscitation, it is essential that emergency physicians understand the principles and practice of endovascular techniques to advocate for the critically unwell patients that may benefit from EVR and be engaged in future implementation.

This practice review aims to provide emergency physicians with an overview of EVR, based around case scenarios of out-of-hospital cardiac arrest and non-compressible haemorrhage. We review the rationale for EVR in these conditions and describe the critical initial step of vascular access. We then describe the physiology, technique, efficacy and safety of three escalating interventions: resuscitative endovascular balloon occlusion of the aorta, selective aortic arch perfusion and extracorporeal cardiopulmonary resuscitation.

## Introduction

 Sustaining a critically-shocked patient through to definitive care is a common and challenging scenario in emergency medicine. A growing number of percutaneous interventions, together termed endovascular resuscitation (EVR), may bridge patients to treatment or recovery through haemostasis, circulatory support or a combination of both. These interventions can be life-saving but may also cause significant harm.

Emergency physicians are most likely to encounter EVR candidates in the context of patients presenting with haemorrhagic shock or in out-of-hospital cardiac arrest (OHCA). Such patients may even present with EVR devices already in-situ, as several services, including critical care teams and dedicated endovascular teams, are employing EVR in the prehospital environment. To best advocate for these patients, it is essential that emergency physicians understand the principles of EVR, the spectrum of utility and complexity involved in different techniques, and their current clinical maturity.

Three interventions will be discussed here: resuscitative endovascular balloon occlusion of the aorta (REBOA), selective aortic arch perfusion (SAAP) and extracorporeal cardiopulmonary resuscitation (ECPR). The crucial preliminary step of vascular access is also described. For this practice review a literature search was conducted relating each device to either haemorrhagic trauma or OHCA, using search terms that can be found in the online supplemental material. Results revealed a small number of key randomised control trials and systematic reviews, reflecting the challenges associated with conducting high-quality research in critically-unwell patients in prehospital and emergency department (ED) settings. We will focus on discussion of key papers that have influenced practice, alongside current and emerging guidelines. We will also discuss evidence from EVR use in other indications, where it informs predictable device performance and the maturity of the technology.

Consider the two ‘pre-alert’ scenarios (boxes 1 and 2): how could endovascular techniques help temporise these patients?

Box 1 **Pre-alert patient 1**

You receive a trauma patient in your emergency department: a young adult pedestrian struck by a heavy goods vehicle. On arrival, the patient is in a state of critical hypovolaemia; heart rate 135 /min, BP 72/40 mm Hg. Examination reveals no airway concern, no chest injuries, overt pelvic disruption (before prehospital pelvic binder placement) and no sign of traumatic brain injury. Following placement of large-bore peripheral cannulae, the patient is transfused two units of packed red cells and two units of thawed plasma. Concurrently, a 5Fr arterial line has been placed in the left common femoral artery under ultrasound guidance through a window cut in the pelvic binder—the invasive BP is 65/45 mm Hg. The patient is too unstable to undergo CT; ultrasound demonstrates no thoracic injury or pericardial tamponade but there is free fluid in the abdomen. While the trauma team continues damage control resuscitation, you are informed that neither the emergency operating theatre nor the interventional radiology suite will be available for at least 30 min.

BP, blood pressure.

Box 2 **Pre-alert patient 2**

You are pre-alerted to the arrival of a 49-year-old male who is in cardiac arrest. He had a witnessed collapse in a nearby shopping centre, with immediate bystander CPR, rapid emergency services presence and subsequent HEMS attendance. On scene he was in VF and has received three shocks and intravenous epinephrine without a ROSC, although he has been demonstrating CPR-induced consciousness. He has been intubated and established on a mechanical CPR device for transfer by road ambulance.

On arrival, he remains in VF despite delivery of two further shocks and intravenous epinephrine and amiodarone. Examination, focused cardiac ultrasound and initial blood gases do not suggest an extracardiac cause for his arrest and his presumed OHCA aetiology is occlusive myocardial infarction. At this point his total low-flow time is approximately 35 min. Your cardiology service will consider intra-arrest PPCI, but there is another patient in the catheterisation suite currently and it will not be available for 30 min.

CPR, cardiopulmonary resuscitation; HEMS, helicopter emergency medical services; OHCA, out-of-hospital cardiac arrest; PPCI, primary percutaneous coronary intervention; ROSC, return of spontaneous circulation; VF, ventricular fibrillation.

## The rationale for endovascular resuscitation

### Out-of-hospital cardiac arrest

In 2023 in England there were 29 625 OHCA where emergency medical services attempted resuscitation, with only 9.0% of patients surviving to discharge. Rates of mortality and neurological disability in survivors have changed little in the last 50 years.^1^ Every minute of cardiac arrest adds to the ischaemic injury burden, with neurologically-intact survival very unlikely beyond 30 min of low-flow.^2^ Patients in OHCA with a shockable rhythm (around 20%) are more likely to survive as arrhythmias commonly result from intracardiac lesions amenable to revascularisation. These patients have the greatest chance of survival if irreversible ischaemia and hypoxia can be avoided.

Conventional advanced life support is limited by four critical factors, all of which could be addressed through the use of EVR. These are (1) the variable reproduction of cardiac output generated by CPR, (2) ineffective circulation of intravenous resuscitation medications along with uncertain efficacies, (3) gauging resuscitation efforts without reliable assessment of flow and pressure (most critically coronary perfusion pressure) and (4) the short time-frame in which to prevent irreversible hypoxic injury, which is often exhausted prehospital.

### Exsanguinating trauma

Haemorrhage is the leading cause of potentially preventable death in both civilian and military trauma.^3 4^ Following the widespread adoption of arterial limb tourniquets, the US military reported an 85% reduction in extremity haemorrhage mortality.^4^ The key remaining cause of death is bleeding in the torso, or non-compressible haemorrhage (NCH), describing the injury of ‘vascular disruption to axial torso vessels, solid organs, pulmonary parenchyma, and/or the bony pelvis, when accompanied by shock’.^5^ Patients with NCH have a high mortality, between 32–86%.^6 7^ Effective management of NCH likely presents the greatest clinical opportunity to improve survival and, with some contraindications, is the target aetiology for trauma EVR.

## Vascular access

Femoral arterial catheterisation is the common and critical first step in delivering EVR. The placement of a vascular access sheath allows rapid haemodynamic monitoring, the delivery of EVR devices and may also be used to isolate a bleeding organ or vascular bed using interventional radiology approaches such as embolisation.

The common femoral artery is preferred because it is readily identified with ultrasound, has sufficient calibre to accommodate vascular access sheaths and is compressible if access fails or following device removal. In severe shock the vascular access sheath may be placed after initial vessel puncture either using ultrasound guidance or, less commonly, surgical cutdown. In patients with pelvic instability, options include cutting a window into the in-situ binder (if material allows) or manual stabilisation with the binder moved toward the feet.

Gaining access is often regarded as the ‘Achilles heel’ of EVR techniques, due to a variety of context-specific challenges. Physical space is often limited by the environment and ongoing resuscitation efforts, leading to suboptimal patient and operator positioning. Body habitus and anatomical variations can complicate ultrasound vessel identification, as can altered haemodynamics caused by intravascular depletion or the use of mechanical CPR devices. To overcome these difficulties a micropuncture device is commonly used, comprising a 21 g echogenic needle, flexible microwire and 4–5 Fr microcatheter sheath. Micropuncture kits are smaller, less traumatic and more easily manipulated than traditional central access kits, yet they allow haemodynamic monitoring and are large enough to pass endovascular device wires. The larger sheaths can then be exchanged using the Seldinger technique. A detailed article on common femoral artery access has been recently published in this journal.^8^

Time is of the essence. With experience, even the most complex techniques can be initiated within 15 min during ongoing CPR in both prehospital and hospital settings^9–11^ but robust training and governance is paramount to ensure rapid, safe and reliable access. In the UK, prehospital placement of arterial catheters by enhanced care teams is increasingly used to guide resuscitation^12^ and to facilitate EVR.^13^

## Resuscitative endovascular balloon occlusion of the aorta

### Physiology and technique

REBOA uses intra-aortic balloon occlusion that can be deployed at different aortic levels. REBOA acts as a functional aortic cross-clamp, providing haemorrhage control distal to the occlusion and increasing afterload and aortic pressure proximal to the occlusion. As coronary blood flow relies on a diastolic pressure gradient between the proximal aorta and right atrium, diastolic blood pressure (BP) is a key factor in cardiopulmonary resuscitation and independently associated with return of spontaneous circulation (ROSC).^14^ Crucially, REBOA can thus increase both cardiac and cerebral perfusion pressures (figure 1A and B, table 1).

**Figure 1:**
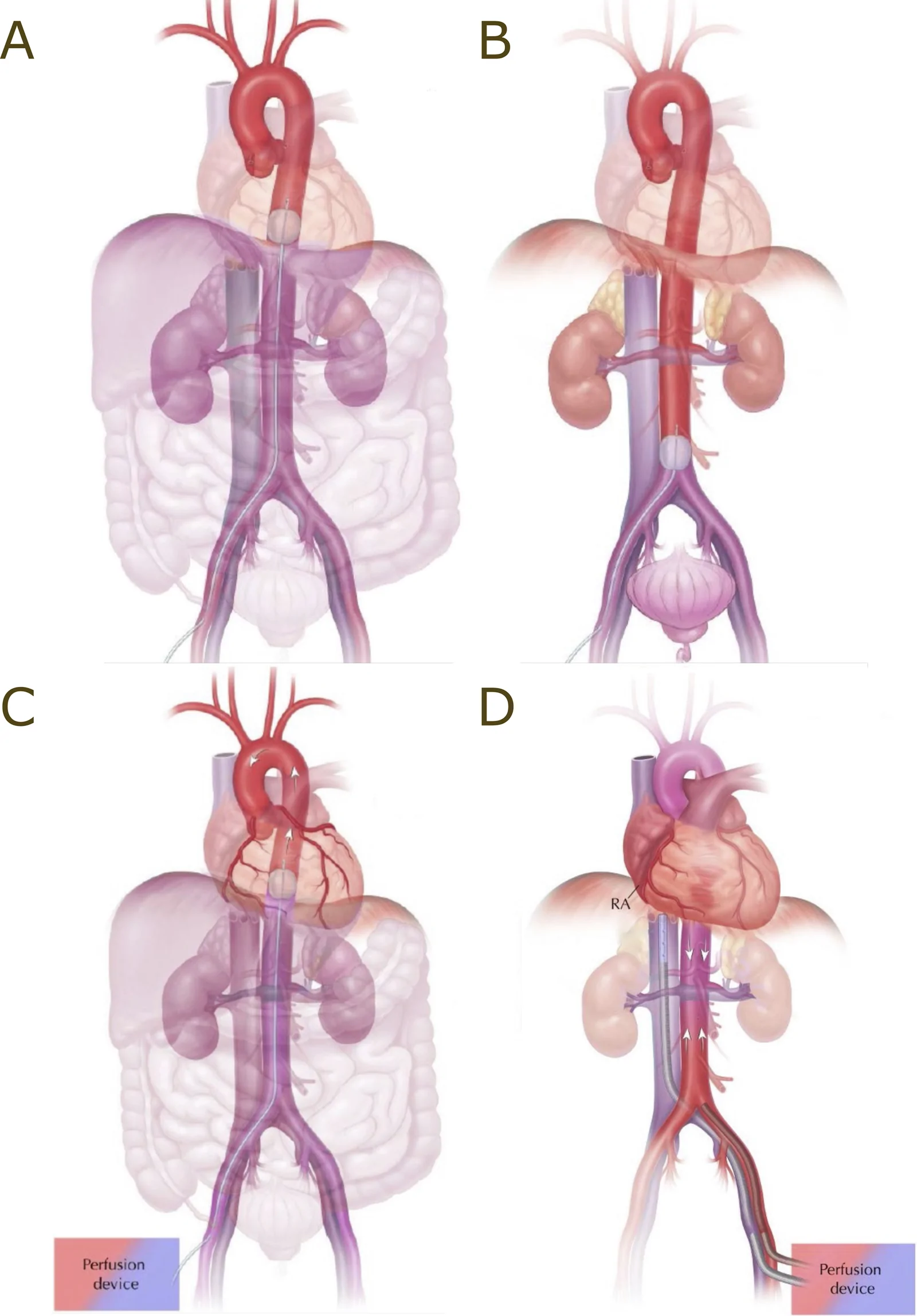
Concepts in endovascular resuscitation. Endovascular resuscitation variably employs aortic occlusion and either regional or systemic perfusion to provide mechanical circulatory support. REBOA uses aortic occlusion to provide distal haemostasis. The balloon can be deployed in either Zone 1 (**A**) or Zone 3 (**B**) depending on the area of suspected bleeding. SAAP (**C**) uses a thoracic aortic balloon occlusion catheter to provide isolated heart and brain perfusion. In ECPR, (**D**) blood is drawn out of the venous circulation at the level of RA by a centrifugal pump and passed through a membrane oxygenator before being returned to the arterial circulation. The point where the reinfused blood meets the antegrade flow from the heart is called the mixing cloud (between arrows). Figure reproduced from Manning *et al*, *J Trauma Acute Care Surg, Emerging hemorrhage control and resuscitation strategies in trauma: Endovascular to extracorporeal, Vol 89 (2S*) with permission from Wolters Kluwer Health. ECPR, extracorporeal cardiopulmonary resuscitation; RA, right atrium; REBOA, resuscitative endovascular balloon occlusion of the aorta; SAAP, selective aortic arch perfusion.

**Table 1:** The endovascular toolkit

Device	Access required	Invasive BP monitoring	Central drug delivery	Distal haemorrhage control	Acute circulatory support	Post-ROSC support
**REBOA**	Femoral artery, 4–8 Fr	Yes	Yes	Yes	Aortic, passive	No
**SAAP**	Femoral artery, 12Fr	Yes	Yes	Yes	Aortic, active	Limited
**ECPR**	Femoral artery (15–19 Fr) and vein (21–25 Fr)	Yes	Yes	No	Whole body, active	Yes

EVR interventions involve varying mechanisms, degrees of vascular access and overall complexity.

BP, blood pressure; ECPR, extracorporeal cardiopulmonary resuscitation; EVR, endovascular resuscitation; REBOA, resuscitative endovascular balloon occlusion of the aorta; ROSC, return of spontaneous circulation; SAAP, selective aortic arch perfusion.

Of the techniques discussed here, REBOA requires the least equipment and smallest catheter (typically 4–8 Fr, figure 2A). The REBOA balloon may be deployed in Zone 1 (Z1—extending from the origin of the left subclavian artery to the coeliac artery, figure 1A), or Zone 3 (Z3—from the lowest renal artery to the aortic bifurcation, figure 1B). Zone 1 REBOA is aimed at haemorrhage caudal to the diaphragm, and Z3 is aimed at providing haemostasis to the pelvic and groin junctional area. The balloon occlusion may be complete, partial or intermittent depending on the device and the perceived balance between the desired effect on physiology and the risks of ongoing occlusion. Complete occlusion (the original technique) offers maximal haemostasis and aortic pressures but confers the greatest risk of ischaemic injury (see below). Intermittent REBOA (iREBOA) is a technique of alternating between full occlusion and distal flow restoration. Partial REBOA (pREBOA) entails dynamic modulation of balloon volume to titrate distal flow against desired effects, using a specific partial occlusion device.

**Figure 2:**
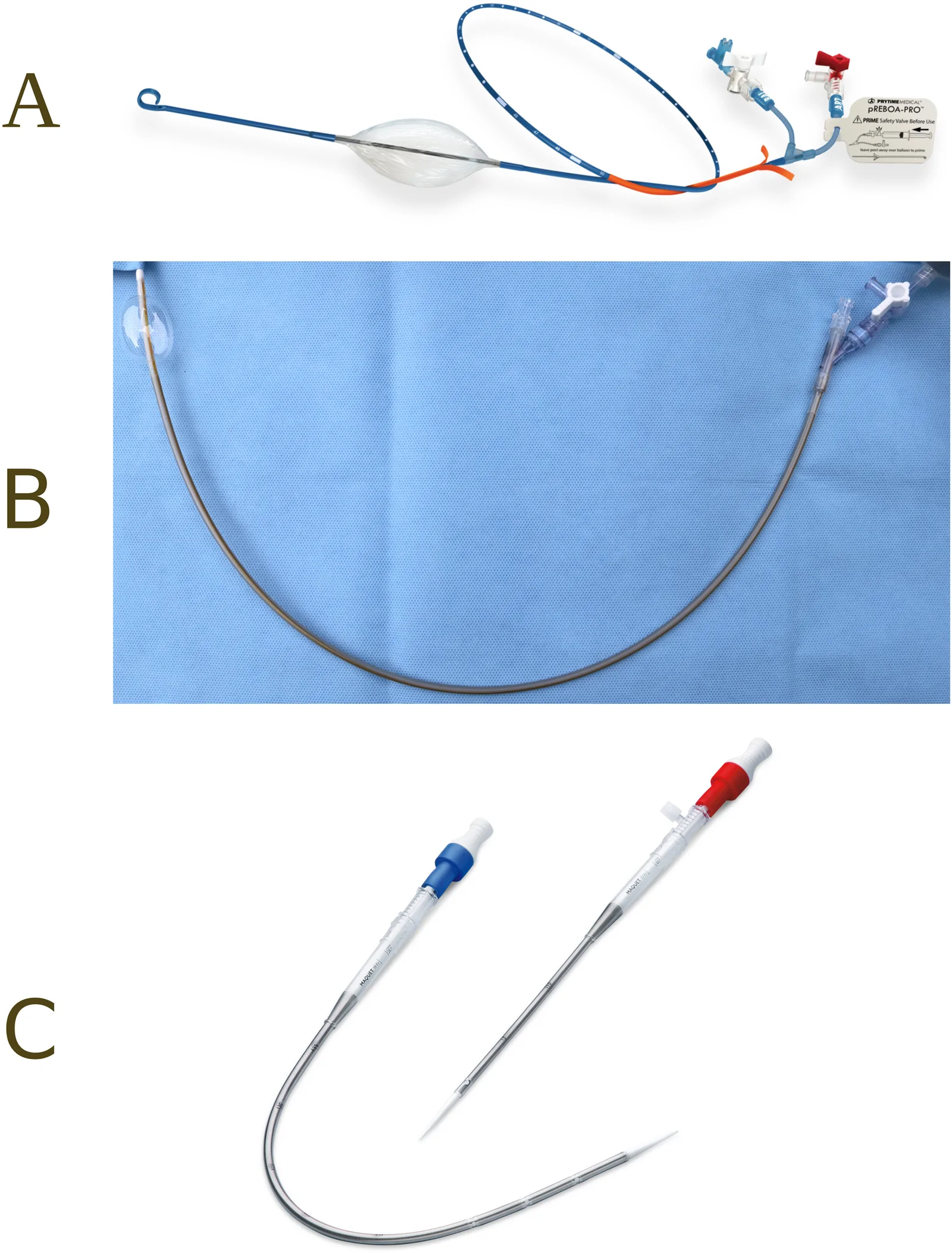
Endovascular resuscitation devices REBOA catheters (**A**, courtesy of Prytime Medical) have a diameter of 4–12 Fr, an occlusive balloon and additional port for invasive pressure monitoring. SAAP catheters (**B**, courtesy of authors) have a larger diameter (up to 12 feet) that permits rapid infusion driven by a perfusion device (not shown). In contrast, ECPR is delivered via large reinforced cannulae (**C**, courtesy of the Getinge Group) sited in the femoral vein and artery and blood passed through an extracorporeal membrane oxygenator (not shown). ECPR, extracorporeal cardiopulmonary resuscitation; REBOA, resuscitative endovascular balloon occlusion of the aorta; SAAP, selective aortic arch perfusion.

### Efficacy

#### REBOA for OHCA

Small studies suggest REBOA may improve coronary perfusion^15^ and increase end-tidal carbon dioxide,^16^ but evidence of improved clinical outcomes is not yet available. Despite being technically and logistically challenging, REBOA for OHCA may be feasible to perform in the prehospital setting.^17^ A UK pilot study of prehospital REBOA for OHCA—the Emergency Resuscitative Endovascular Balloon Occlusion of the Aorta in Out of Hospital Cardiac Arrest (ERICA-ARREST) trial (NCT06071910)—is currently recruiting.^18^

A Norwegian multicentre, randomised phase 2 trial of prehospital REBOA versus standard advanced life support (ALS) in OHCA has concluded recruitment and is in analysis at the prepublication stage. This trial, the Resuscitative Endovascular Balloon Occlusion of the Aorta in Non-traumatic Out of Hospital Cardiac Arrest (REBOARREST) trial (NCT04596514) has a primary endpoint of proportion of subjects achieving ROSC.^19^

#### REBOA for trauma

Between 2011–2014, large animal translational studies demonstrated that REBOA in haemorrhage significantly increased BP and survival, caused less physiological derangement than resuscitative thoracotomy and aortic clamping (the alternative intervention), and increased brain perfusion compared with controls. Single-centre studies have variably reported non-inferiority with regard to patient outcomes but the most comprehensive meta-analysis, of 11 observational studies and 5866 patients, found no benefit to REBOA when compared with standard care.^20^

The only randomised controlled trial (RCT) to date provides valuable information and demonstrates the challenges of pragmatically implementing EVR. The UK-REBOA trial was a multicentre, Bayesian, group-sequential trial comparing ED-initiated REBOA to standard care in UK major trauma centres.^21^ 90 patients with suspected exsanguinating haemorrhage received either REBOA sited in the ED (n=46) or standard care (n=44). At 90 days, 25 of 46 patients (54%) had died in the REBOA arm versus 18 of 43 patients (42%) in the standard care group (OR 1.58 (95% credible interval, 0.72 to 3.52). The posterior probability of an OR >1 suggesting increased odds of death with REBOA was 86.9%. The trial was stopped early as interim analysis of prespecified limits for harm had been met.

Studying this extremely sick population (who had a median injury severity score of 41) was an impressive feat. While the authors conclude that REBOA did not improve survival, there are major caveats around timing, training and decision-making. REBOA was performed in-hospital, a considerable time after initial injury, in a healthcare system unfamiliar with EVR. Most participating centres had not used REBOA prior to this study, and there is a clear association between centre volume and outcomes.^22^ Only 19 of the REBOA group received aortic occlusion, which took a median of 32 min to perform, whereas other studies report less than 15 min.^17 23^ The decision to use REBOA may have been made more difficult as most subjects had complex blunt trauma without a clear focus for haemorrhage.

Despite these data, the US Trauma Quality Improvement Programme registry reports steady utilisation of REBOA in several high-volume Level 1 trauma centres, with 3398 REBOA deployments (predominantly in Zone 3 and for blunt trauma) between 2017–2022.^24^ There is furthermore continued interest in REBOA in a military context, where access to established measures such as blood product resuscitation and damage control surgery may be severely limited.

We speculate that the dissonance between evidence and adoption may reflect a belief among practitioners, based on their own centre experience, that the risks of life-threatening complications and potential delays to definitive treatment can be surmounted through training and a cohesive ‘door-to-discharge’ strategy.

#### Emerging research

Given the brief window for intervention in exsanguinating trauma, there is a current focus on moving REBOA closer to the point of injury, alongside technical modifications to reduce harmful effects of aortic occlusion. Partial REBOA, describing titrated balloon deflation to allow distal flow without total ischaemia, appears feasible in the UK prehospital setting.^25^

### Safety

The main risk from REBOA is ischaemia distal to the balloon (eg, liver, renal, bowel, spinal cord) and potential compromise to limb perfusion, secondary to stasis and subsequent thrombus formation distal to the balloon and around the vascular sheath.^26^ The increase in cardiac afterload might potentially worsen myocardial ischaemia, induce left ventricular (LV) dilatation and cause pulmonary oedema.^27^ Haemorrhage arising from vessels proximal to balloon inflation (eg, pericardial tamponade, haemothorax) may be exacerbated. Balloon deflation and reperfusion releases lactate, hydrogen ions and reactive oxygen species from ischaemic tissues causing a systemic inflammatory response that compounds existing injuries and can cause de novo organ failure. Iatrogenic vascular injury can occur along the path of the catheter, or due to inadvertent extra-aortic device deployment. Repeated instrumentation attempts can cause dissection and damage branch bifurcations. Needle punctures, notably those more proximal, may not be amenable to direct pressure.

Therefore, emergency physicians should closely monitor the access site and ipsilateral limb, document and minimise total occlusion time, and maintain clear situational awareness of balloon status, including any planned or completed deflation times.

## Selective aortic arch perfusion

### Physiology and technique

SAAP is an experimental extracorporeal perfusion technique that uses a thoracic aortic balloon occlusion catheter with a large infusion lumen to provide isolated heart and brain perfusion. The thoracic aorta is occluded just above the diaphragm (in Zone 1) and an oxygenated perfusate is delivered to the vessels of the thoracic aorta, including the coronaries and aortic arch vessels (figure 1C, table 1). Medications, such as epinephrine, may also be delivered above the balloon occlusion. SAAP uses the same membrane oxygenator employed in extracorporeal membrane oxygenation (ECMO).

No SAAP catheters are currently approved for clinical use, but its combination of aortic occlusion and limited perfusion is conceptually important and an appealing future device. While SAAP was developed to maximise cardiocerebral perfusion during non-traumatic resuscitation, its potential to control subdiaphragmatic haemorrhage and permit rapid volume replacement mean future use may include haemorrhagic shock and haemorrhage-induced traumatic cardiac arrest (TCA).

The SAAP catheter (figure 2B) is advanced from a femoral artery to the Z1 REBOA position, and the balloon inflated. An infusion of oxygenated perfusate (blood, perfluorocarbon or haemoglobin-based oxygen carrier, HBOC) can then be delivered in a retrograde fashion to the aortic arch via the catheter lumen at around 10 mL/kg/min. The combination of aortic occlusion and rapid retrograde infusion aims to initially close the aortic valve and perfuse the coronary ostia and aortic arch vessels. As cardiac function improves, the aortic valve will reopen and SAAP perfusion can be reduced.

The SAAP catheter may be deployed primarily for initial aortic occlusion, permitting a trial of REBOA prior to escalating to active circulatory support. If the initial rescue infusion of oxygenated perfusate is not successful at achieving ROSC, femoral venous access could be obtained and venous blood oxygenated and recirculated, in essence offering veno-arterial ECMO (VA-ECMO) limited to the aortic arch.

### Efficacy

#### SAAP for OHCA

In large-animal models, SAAP increases coronary perfusion pressure and reduces time to ROSC in ventricular fibrillation (VF) arrest.^28^ Coinfusion of epinephrine via the SAAP catheter may further increase cerebral perfusion pressure (CPP) and appears more effective than peripherally-infused epinephrine.^29^ SAAP has recently been demonstrated in a cadaveric human model, alongside angiography via the SAAP catheter, which could permit concomitant salvage PPCI.^30^

#### SAAP for trauma

Preclinical porcine studies of SAAP in uncontrolled haemorrhage have reported favourable physiological effects using whole blood and HBOC perfusates.^31 32^ In a head-to-head comparison with REBOA, ROSC was achieved in 6/10 treated with SAAP and Lactated Ringer’s solution as perfusate, and in 10/10 treated with SAAP using whole blood, whereas none of the animals managed with REBOA plus simultaneous peripheral blood transfusion achieved ROSC.^33^

These studies suggest that SAAP with oxygenated perfusates promote ROSC and are superior to non-oxygen carrying crystalloid solutions.^34^ SAAP with whole blood is planned for initial clinical study, given that no HBOC is presently approved for use. Recent evidence suggests the whole-blood SAAP can achieve ROSC after up to 15 min of haemorrhagic arrest and point to a potential role in refractory TCA.^35^ The first clinical data on the use of SAAP are awaited. The provision of aortic perfusion devices is commonly limited to hospitals with cardiothoracic services, which are not always co-located with emergency or trauma services to which candidate patients will present. This is likely to limit the number of centres that could feasibly offer SAAP.

### Safety

SAAP is more technically and logistically challenging than REBOA, requiring a larger-bore (12Fr) femoral catheter, increasing the risk of vascular complications. The risks of aortic occlusion mirror those of REBOA. Aortic arch perfusion can cause volume overload with pulmonary congestion and flow must be titrated carefully. The utmost care must be taken to avoid air emboli.

## Extracorporeal cardiopulmonary resuscitation

### Physiology and technique

VA-ECMO is a complex technique used to provide partial or total cardiorespiratory support through oxygenating and removing carbon dioxide from autologous blood extracted from the venous circulation and returned to the arterial circulation. ECPR refers specifically to the use of peripheral VA-ECMO in refractory cardiac arrest. Other uses and configurations of VA-ECMO will not be discussed in this review.

During ECPR, large-bore ECMO cannulae are advanced into both the femoral artery (15–19F) and vein (21–25F) to sit in the distal aorta and supradiaphragmatic vena cava respectively. The cannulae (figure 2C) are connected to a primed anticoagulant-bonded extracorporeal circuit. Blood is drawn out of the vena cava by a centrifugal pump and passes through a membrane oxygenator before being returned to the arterial circulation under positive pressure. Reinfused blood from the ECMO circuit flows retrograde to perfuse the aorta and its branches. Extracorporeal blood flow is increased to replace approximately 60–80% of the cardiac output. Systemic anticoagulation is often used to reduce the risk of circuit thrombosis. To avoid ischaemia in the cannulated limb, a distal perfusion cannula is usually placed in the superficial femoral artery to provide antegrade flow. Unlike REBOA and SAAP, which are very time-limited, patients can receive support from VA-ECMO for many days, allowing more time for myocardial recovery (table 1).

### Efficacy

#### ECPR for OHCA

Two recent RCTs comparing ECPR to standard care suggest improved survival with favourable neurological outcomes. In the single-centre ARREST trial, Yannopoulos *et al* compared ECPR (n*=*15) to ALS (n*=*15) for refractory VF/ventricular tachycardia (VT) OHCA, reporting significant improvements in survival to discharge (43% vs 7%).^9^ The study was terminated prematurely at interim analysis due to strong evidence of benefit. In Prague, Belohlavek and colleagues randomised 256 patients with OHCA with any initial rhythm to either early intra-arrest transport, cooling and catheterisation-lab ECPR followed by PPCI (n*=*124), or ALS on site and PPCI transfer on ROSC (n=132).^10^ ECPR recipients had significantly better 30-day neurological outcomes (30.6% vs 18.2%) but not 180-day survival with good neurological outcome (the primary endpoint, 31.5% vs 22%). Importantly, there were high rates of patient crossover from control to ECPR arm (and subsequent survival), favouring the control arm in the intention-to-treat analysis. These two studies suggest a survival benefit for ECPR use in OHCA, supported further by a meta-analysis of these data and several propensity-matched studies.^36^ Notably however, these were single-centre studies involved very experienced teams and mature ECPR systems, in effect under ‘optimal conditions’, shown by the fact that median times from ED arrival to ECMO flow were only 7 and 10 min, respectively.

The INCEPTION trial (Early Initiation of Extracorporeal Life Support in refractory OHCA) studied whether ECPR could be effective for OCHA under ‘real-world’ conditions within existing systems. INCEPTION was a pragmatic RCT of ECPR (n=70) versus standard ALS (n=64) in refractory VF/VT OHCA across multiple centres in the Netherlands.^37^ In contrast to the ARREST and Prague studies, participating hospitals did not follow a rigorous trial protocol and had relatively little experience of providing ECPR for OHCA. No significant difference in primary outcome (survival with a favourable neurological outcome at 30 days) was shown (20% vs 16%) and survival in the ECPR group (9.6%) was notably lower than other trials and case series. Median time to ECPR flow was much longer than (36 min from ED and 74 min total low-flow)—probably beyond that compatible with neurologically intact survival^11^—and the ECPR group a median hospital stay of only 2 days. These are important caveats, as an analysis of 7488 ECPR patients from the Extracorporeal Life Support Organization(ELSO) registry suggest survival is associated with centre volume and specialist post-cannulation care.^38^ As with the UK-REBOA trial for REBOA in haemorrhagic trauma, INCEPTION demonstrates the challenges associated with de novo implementation of EVR.

Taken together, a meta-analysis of four RCTs suggests that ECPR may increase neurologically-intact survival from OHCA, and a Bayesian meta-analysis found a 71.1% and 75.8% posterior probability of a clinically-relevant ECPR-based treatment effect on 6-month neurologically favourable survival in patients with all rhythms and shockable rhythms respectively.^36 39^ However, the total number of patients studied remains small (n=433) and the importance of time to cannulation, system-wide adjustments and high centre volume for success appear significant barriers to making ECPR widely accessible.

#### ECPR for trauma

ECPR is not a viable option in the scenario of uncontrolled bleeding, but it may be considered for cardiogenic shock either secondary to direct cardiac contusion, or for myocardial dysfunction following initial damage control surgery. For the patient in TCA, ECMO may have a role as part of an emergency preservation and resuscitation (EPR) strategy, where it is used rapidly to cool the patient to 10 degrees Celsius, and induce hypothermic organ preservation while surgical haemostasis is attempted. Large volumes of ice-cold saline are delivered to the aorta, via femoral access or direct to the arch, and may be recirculated via a central venous catheter. This rapid cooling technique is more akin to transplant organ preservation than the graded hypothermia extensively studied in the postarrest population. However, while cooling to near-zero Celsius dramatically reduces cellular metabolism, cold ischaemia in hypo-osmolar solutions causes cellular oedema and acidosis, and impaired enzymatic coagulation and platelet function may worsen trauma coagulopathy. Preclinical studies suggest that animals can survive neurologically intact following extended periods of EPR after TCA.^40 41^ The first clinical trial on EPR for cardiac arrest in trauma is currently enrolling at the University of Maryland Shock Trauma Centre in Baltimore.^42^

#### Emerging research

In most scenarios prehospital initiation of ECPR should offer more rapid establishment of full support than expedited transfer to a waiting in-hospital ECPR team. Prospective registry analyses from the experienced Parisian group suggest prehospital ECPR initiation is associated with both greater survival and more favourable neurological outcomes versus in-hospital.^11^ There is much focus on development of prehospital ECPR services, with feasibility reported in numerous countries and localities.^43^ Most recently, the London-based SUB30 and Australian PRECARE (Pre-hospital ECPR for Refractory Cardiac Arrest) trials demonstrated safe and effective delivery of ECPR to patients with OHCA.^44 45^ The ON-SCENE (on-scene initiation of ECPR during refractory out-of-hospital cardiac arrest) trial, a Dutch multicentre stepped-wedge study of prehospital ECPR versus standard care, finished recruitment in July 2025 and will report on efficacy and cost-effectiveness.^46^

### Safety

More complex circuitry and prolonged time ‘on pump’ increases the potential for complications compared with REBOA and SAAP. Haemorrhage is the most common major complication, both ‘local’ to the cannulation site and in ‘remote’ intracerebral, gastrointestinal and retroperitoneal spaces.^47^ Larger femoral cannulae increase the likelihood of vascular injury during cannulation. The ECMO circuit may fail or inadvertently become disconnected during assessment or transfer.

If ROSC is achieved, the ECPR patient has a dual circulation with native cardiac output and retrograde ECMO flow that meet in a ‘mixing cloud’ (figure 1D) leading to unique complications. Poor gas exchange in the lung or membrane oxygenator can lead to hypoxaemia in the body region supplied by the affected circulation, termed differential oxygenation (formally harlequin or north/south syndrome).^48^ For example, poor pulmonary gas exchange will lead to hypoxaemic perfusion of the heart, brain and upper limbs. Saturation measurements from the right arm are therefore paramount to recognise differential oxygenation. Retrograde aortic flow may impair aortic valve opening or exacerbate aortic incompetence, causing LV distension, myocardial ischaemia and pulmonary oedema.^49^

ECPR should only be provided by appropriately-trained, skilled teams with highly-developed procedures for cannulation, ECMO initiation, safe transfer and pre-agreed direct access to cardiac catheterisation and cross-sectional imaging facilities.

## EVR in action: current guidelines on OHCA and trauma

### Out-of-hospital cardiac arrest

ECPR is currently the best-evidenced EVR strategy for OHCA and the only therapy to feature in international guidelines. The 2024 International Liaison Committee On Resuscitation and 2025 European Resuscitation guidelines take into account recent trial data and suggest that ECPR may be considered for ‘select adults’ in refractory OHCA where conventional CPR is failing and in settings where this can be implemented, with a weak recommendation.^50 51^ ‘Selection’ here refers largely to inclusion criteria derived from major studies, with an emphasis on the possibility of a reversible pathology and known low-flow time, as time from arrest to ECPR is an independent predictor of survival (table 2).^11 52^ There is emphasis on prompt recognition of refractory cardiac arrest, rapid activation of the ECPR team and the consideration of the effect of ECPR on neuroprognostication and organ donation, highlighting how successful ECPR interacts with the entire chain of survival.^53^

**Table 2:** Typical selection criteria for endovascular resuscitation

	Medical	Trauma
Source	*ERC 2025 Guidelines on ECPR* ^50^	*Joint USA Consensus Statements on REBOA* ^55 56^
Selection criteria	Absolute: Witnessed OHCA with immediate CPR. Upper age limit 50–75 without frailty or major comorbidities. Three consecutive unsuccessful defibrillation attempts or >10 min of resuscitation. Relative: Known or suspected treatable underlying cause. Expected cannulation <60 min after OHCA. Initial shockable or PEA rhythm. ROSC at any time prior to cannulation. Presence of signs of life during CPR. ETCO2 >1.3 kPa.	Absolute: Suspected life-threatening haemorrhage below the diaphragm. Definitive haemostasis available within 15 min of Zone 1 aortic occlusion. Relative: Definitive haemostasis available within 60 min of Zone 3 aortic occlusion. Absence of thoracic haemorrhage or pericardial tamponade.

Appropriate patient selection is critical to successful use of endovascular devices, but decision-makers need to act rapidly and with often with incomplete information. The above criteria, derived from European and American guidelines and consensus statements, typify absolute and relative selection criteria for EVR in medical and trauma scenarios. Crucially however, institutional experience will also guide local practice.

CPR, cardiopulmonary resuscitation; ECPR, extracorporeal cardiopulmonary resuscitation; ERC, European Resuscitation Council; ETCO2, end tidal carbon dioxide; EVR, endovascular resuscitation; OHCA, out-of-hospital cardiac arrest; PEA, pulseless electrical activity; REBOA, resuscitative endovascular balloon occlusion of the aorta; ROSC, return of spontaneous circulation.

### Trauma

For haemorrhagic shock, only REBOA is currently included in trauma guidelines.^54^ A 2019 consensus statement from the USA suggests that it may be used for traumatic life-threatening haemorrhage below the diaphragm in patients in haemorrhagic shock who are refractory to resuscitation, but only in settings where definitive haemorrhage control will soon be available. It is emphasised that every effort should be made to minimise aortic occlusion times, and that complete aortic occlusion at the distal thoracic aorta should be limited to 15 min, and more distal occlusion limited to less than 30–60 min.^55^ Suspected thoracic haemorrhage is an absolute contraindication to use (table 2). This guidance predates important information from UK-REBOA, and technological developments such as iREBOA and pREBOA.

## Conclusions

Endovascular therapies are simple in concept but complex in execution. To maintain any potential benefit from the initial lifesaving procedure, EVR likely requires an unbroken sequence of rapid primary decision-making, time-critical device deployment and withdrawal, and specialist ongoing medical and surgical care. This complexity makes EVR difficult to study outside of mature systems.

As survival following OHCA and NCH remains so low, EVR adoption appears likely to continue in high-volume metropolitan centres with existing EVR programmes. Further research is required to determine whether positive results in isolated systems—such as for ECPR in OHCA—can be implemented more widely, and to determine which patient subgroups may benefit most.

When considering EVR for their patient, clinicians should ask themselves—will EVR be delivered to the right patient, in a timely manner, by experienced practitioners, in a system where it is integrated into care pathways and institutional practice through and beyond intensive care? If so, EVR may be beneficial; if not, it is likely to cause delay or harm.

## Supplementary material

10.1136/emermed-2025-215376 online supplemental file 1
