# Supplementary material for: Endovascular resuscitation: an expert practice review
Source: Emerg Med J. 2026 Apr 30;43(10):e215376. doi: 10.1136/emermed-2025-215376 (PMC13638346; doi:10.1136/emermed-2025-215376)
Supplement: Supplementary file 1 [file emermed-43-10-s001.docx]

# SUPPLEMENTARY MATERIAL 1: SEARCH TERMS

1. REBOA in haemorrhagic shock and traumatic cardiac arrest

(REBOA OR "resuscitative endovascular balloon occlusion" OR "endovascular balloon occlusion of the aorta" OR "aortic balloon occlusion" OR "intra-aortic balloon occlusion" OR "resuscitative aortic occlusion" OR "endovascular aortic occlusion") AND (hemorrhag* OR haemorrhag* OR "hemorrhagic shock" OR "haemorrhagic shock" OR "hypovolemic shock" OR "hypovolaemic shock" OR "traumatic shock" OR exsanguination OR "blood loss" OR "massive hemorrhage" OR "massive haemorrhage" OR "severe bleeding" OR "life-threatening hemorrhage" OR "life-threatening haemorrhage" OR "uncontrolled bleeding" OR "uncontrolled hemorrhage" OR "uncontrolled haemorrhage") AND (adult* OR "18 years" OR "over 18" OR "aged 18") NOT (pediatric* OR paediatric* OR child* OR infant* OR neonat* OR adolescent*)

2. REBOA in OHCA

(REBOA OR "resuscitative endovascular balloon occlusion" OR "endovascular balloon occlusion of the aorta" OR "aortic balloon occlusion" OR "intra-aortic balloon occlusion" OR "resuscitative aortic occlusion" OR "endovascular aortic occlusion") AND ("out-of-hospital cardiac arrest" OR OHCA OR "out of hospital cardiac arrest" OR "prehospital cardiac arrest" OR "pre-hospital cardiac arrest" OR ("cardiac arrest" AND ("out-of-hospital" OR prehospital OR "pre-hospital" OR community)) OR ("sudden cardiac arrest" AND ("out-of-hospital" OR prehospital OR community)) OR ("cardiopulmonary arrest" AND ("out-of-hospital" OR prehospital))) AND (adult* OR "18 years" OR "over 18" OR "aged 18") NOT (pediatric* OR paediatric* OR child* OR infant* OR neonat* OR adolescent*)

3. SAAP in haemorrhagic shock and traumatic cardiac arrest

(SAAP OR "selective aortic arch perfusion" OR "selective aortic perfusion" OR "aortic arch perfusion" OR "targeted aortic perfusion" OR ("cerebral perfusion" AND aortic)) AND (hemorrhag* OR haemorrhag* OR "hemorrhagic shock" OR "haemorrhagic shock" OR "hypovolemic shock" OR "hypovolaemic shock" OR "traumatic shock" OR exsanguination OR "blood loss" OR "massive hemorrhage" OR "massive haemorrhage" OR "severe bleeding" OR "life-threatening hemorrhage" OR "life-threatening haemorrhage" OR "uncontrolled bleeding" OR "uncontrolled hemorrhage" OR "uncontrolled haemorrhage") AND (adult* OR "18 years" OR "over 18" OR "aged 18") NOT (pediatric* OR paediatric* OR child* OR infant* OR neonat* OR adolescent*)

4. SAAP in OHCA

(SAAP OR "selective aortic arch perfusion" OR "selective aortic perfusion" OR "aortic arch perfusion" OR "targeted aortic perfusion" OR ("cerebral perfusion" AND aortic)) AND ("out-of-hospital cardiac arrest" OR OHCA OR "out of hospital cardiac arrest" OR "prehospital cardiac arrest" OR "pre-hospital cardiac arrest" OR ("cardiac arrest" AND ("out-of-hospital" OR prehospital OR "pre-hospital" OR community)) OR ("sudden cardiac arrest" AND ("out-of-hospital" OR prehospital OR community)) OR ("cardiopulmonary arrest" AND ("out-of-hospital" OR prehospital))) AND (adult* OR "18 years" OR "over 18" OR "aged 18") NOT (pediatric* OR paediatric* OR child* OR infant* OR neonat* OR adolescent*)

5. ECPR in haemorrhagic shock and traumatic cardiac arrest

(ECPR OR "extracorporeal cardiopulmonary resuscitation" OR "extracorporeal CPR" OR "E-CPR" OR (("extracorporeal life support" OR ECLS OR "extracorporeal membrane oxygenation" OR ECMO OR "veno-arterial ECMO" OR "VA-ECMO") AND (hemorrhag* OR haemorrhag*))) AND (hemorrhag* OR haemorrhag* OR "hemorrhagic shock" OR "haemorrhagic shock" OR "hypovolemic shock" OR "hypovolaemic shock" OR "traumatic shock" OR exsanguination OR "blood loss" OR "massive hemorrhage" OR "massive haemorrhage" OR "severe bleeding" OR "life-threatening hemorrhage" OR "life-threatening haemorrhage" OR "uncontrolled bleeding" OR "uncontrolled hemorrhage" OR "uncontrolled haemorrhage") AND (adult* OR "18 years" OR "over 18" OR "aged 18") NOT (pediatric* OR paediatric* OR child* OR infant* OR neonat* OR adolescent*)

6. ECPR in OHCA

(ECPR OR "extracorporeal cardiopulmonary resuscitation" OR "extracorporeal CPR" OR "E-CPR" OR ("extracorporeal life support" AND "cardiac arrest") OR ("ECLS" AND "cardiac arrest") OR ("extracorporeal membrane oxygenation" AND "cardiac arrest") OR ("ECMO" AND "cardiac arrest" AND resuscitation) OR ("veno-arterial ECMO" AND "cardiac arrest") OR ("VA-ECMO" AND "cardiac arrest")) AND ("out-of-hospital cardiac arrest" OR OHCA OR "out of hospital cardiac arrest" OR "prehospital cardiac arrest" OR "pre-hospital cardiac arrest" OR ("cardiac arrest" AND ("out-of-hospital" OR prehospital OR "pre-hospital" OR community)) OR ("sudden cardiac arrest" AND ("out-of-hospital" OR prehospital OR community)) OR ("cardiopulmonary arrest" AND ("out-of-hospital" OR prehospital))) AND (adult* OR "18 years" OR "over 18" OR "aged 18") NOT (pediatric* OR paediatric* OR child* OR infant* OR neonat* OR adolescent*)
